# Correction to: SPEAQeasy: a scalable pipeline for expression analysis and quantification for R/bioconductor‑powered RNA‑seq analyses

**DOI:** 10.1186/s12859-021-04283-5

**Published:** 2021-07-22

**Authors:** Nicholas J. Eagles, Emily E. Burke, Jacob Leonard, Brianna K. Barry, Joshua M. Stolz, Louise Huuki, BaDoi N. Phan, Violeta Larios Serrato, Everardo Gutiérrez-Millán, Israel Aguilar-Ordoñez, Andrew E. Jaffe, Leonardo Collado-Torres

**Affiliations:** 1 Lieber Institute for Brain Development, Johns Hopkins Medical Campus, Baltimore, MD 21205 USA; 2 Winter Genomics, Salaverry 874 int 100, Lindavista, CDMX 07300 Mexico; 3 QuestBridge Scholar, Palo Alto, CA 94303 USA; 4 Department of Neuroscience, Johns Hopkins School of Medicine, Baltimore, MD 21205 USA; 5 Computational Biology Department, School of Computer Science, Carnegie Mellon University, Pittsburgh, PA 15213 USA; 6 Medical Scientist Training Program, School of Medicine, University of Pittsburgh, Pittsburgh, PA 15213 USA; 7 Instituto Politécnico Nacional, Escuela Nacional de Ciencias Biológicas,, Mexico City, CDMX 11340 Mexico; 8 Department of Supercomputing, Instituto Nacional de Medicina Genómica (INMEGEN), Mexico City, CDMX 14610 Mexico; 9 Center for Computational Biology, Johns Hopkins University, Baltimore, MD 21205 USA; 10 Department of Biostatistics, Johns Hopkins Bloomberg School of Public Health, Baltimore, MD 21205 USA; 11 Department of Genetic Medicine, McKusick-Nathans Institute of Genetic Medicine, Johns Hopkins University School of Medicine, Baltimore, MD 21205 USA; 12 Department of Psychiatry and Behavioral Sciences, Johns Hopkins School of Medicine, Baltimore, MD 21205 USA; 13 Department of Mental Health, Johns Hopkins Bloomberg School of Public Health, Baltimore, MD 21205 USA

## Correction to: BMC Bioinformatics (2021) 22:224 10.1186/s12859-021-04142-3

Following the publication of the original article [1], the authors identified the incorrect published Additional file 4 and missing Additional file 1: figures S1 and Additional file 2: S2.

The original article [1] has been corrected.

## Supplementary Information

**Additional file 1**: **Figure S1**. Expected vs. Actual ERCC concentration. SPEAQeasy produces plots for each sample, for easy visual comparison of expected ERCC transcript abundance with the kallisto-measured concentration.

**Additional file 2**: **Figure S2**. SPEAQeasy logs tracing computational steps by sample. To aid transparency and greatly simplify the source of execution errors, SPEAQeasy automatically generates logs with several pieces of information for every sample. In order of submission, the name of each Nextflow process is printed, along with (1) the working directory: where all relevant files are present, (2) the exit code: a standard indication of whether the process succeeded or how it failed, (3) a list of the specific commands run during the given process. Above is a snapshot of the top of an example log

**Additional file 4**: SNVs supplementary BED files. The common SNVs used for sample identification are stored in the BED files (**A**) common_missense_SNVs_hg19.bed and (**B**) common_missense_SNVs_hg38.bed.

**Additional file 4**: SNVs supplementary BED files. The common SNVs used for sample identification are stored in the BED files (**A**) common_missense_SNVs_hg19.bed and (**B**) common_missense_SNVs_hg38.bed.
