# Supplementary figures and images for: SPEAQeasy: a scalable pipeline for expression analysis and quantification for R/bioconductor-powered RNA-seq analyses
Source: BMC Bioinformatics. 2021 May 1;22:224. doi: 10.1186/s12859-021-04142-3 (PMC8088074; doi:10.1186/s12859-021-04142-3)

# Sample\_2914

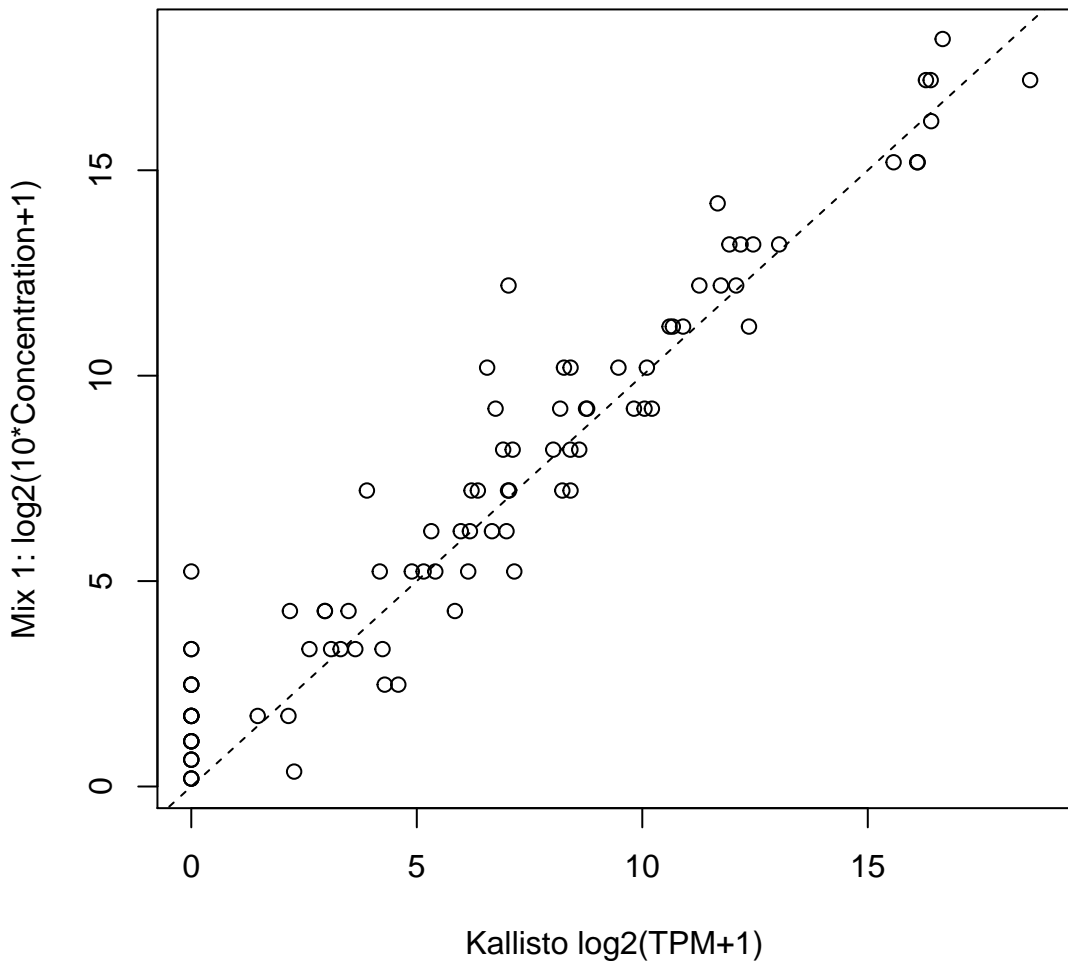

Supplement: Supplementary file 1 — Additional file 1. Figure S1: Expected vs. Actual ERCC concentration. SPEAQeasy produces plots for each sample, for easy visual comparison of expected ERCC transcript abundance with the kallisto-measured concentration. [file 12859_2021_4142_MOESM1_ESM.pdf]

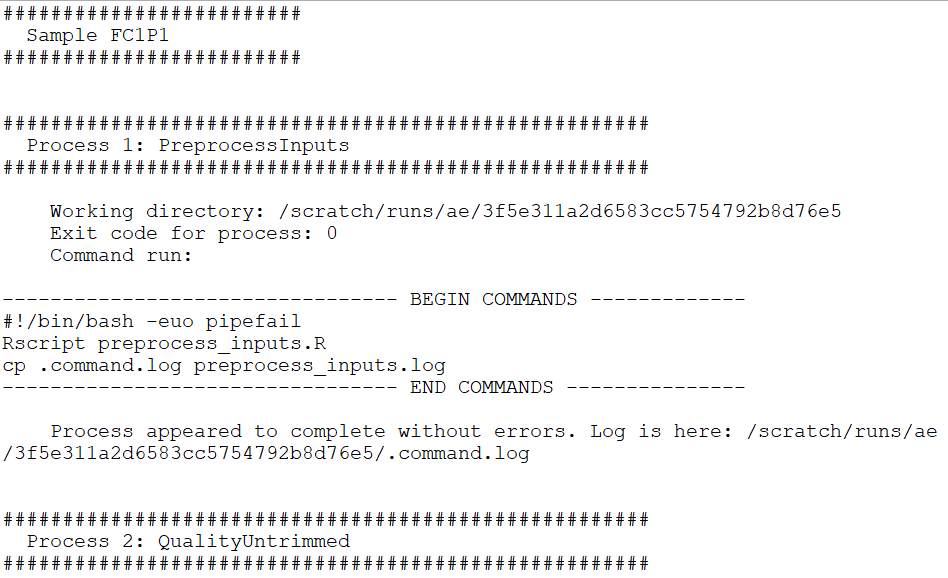

Supplement: Supplementary file 2 — Additional file 2: Figure S2: SPEAQeasy logs tracing computational steps by sample. To aid transparency and greatly simplify the source of execution errors, SPEAQeasy automatically generates logs with several pieces of information for every sample. In order of submission, the name of each Nextflow process is printed, along with (1) the working directory: where all relevant files are present, (2) the exit code: a standard indication of whether the process succeeded or how it failed, (3) a list of the specific commands run during the given process. Above is a snapshot of the top of an example log [file 12859_2021_4142_MOESM2_ESM.png]
